# Isolated Chromosome 6q27 Terminal Deletion Syndrome

**DOI:** 10.7759/cureus.8103

**Published:** 2020-05-13

**Authors:** Sabita Bhatta, Marsha Medows, Yogesh Acharya

**Affiliations:** 1 Pediatrics, Woodhull Medical Center, New York, USA; 2 Pediatrics, Woodhull Hospital Center, Brooklyn, USA; 3 Pediatrics, New York University School of Medicine, New York, USA; 4 Vascular and Endovascular Surgery, Western Vascular Institute, Galway, IRL

**Keywords:** chromosome 6, chromosome deletion, chromosome 6q27 terminal deletion, syndrome, congenital, seizure, facial asymmetry, developmental delay, child

## Abstract

Any change in either the short (p) or long (q) arm of chromosome six can result in a variety of disorders.

A two-year-old female child came to us with a history of sudden onset generalized tonic-clonic seizure. She had a syndromic face with frontal bossing and palpable thinning of the right lower lip and an apparent facial asymmetry while crying due to the hypoplasia of the right depressor angularis oris. Her joints were hypermobile and hypotonic. Chromosomal karyotyping exhibited a normal female karyotype, but pathogenic microarray genetic evaluation showed a loss of approximately 783 kb of the 6q27 terminus. She was diagnosed with chromosome 6q27 terminal deletion and managed with anti-seizure medications.

Chromosome 6q27 terminal deletion can present with an array of structural and developmental anomalies. It is, therefore, necessary to understand the typical phenotypic and distinctive clinical features of congenital chromosome 6q27 terminal deletion syndrome for early diagnosis and intervention.

## Introduction

Multiple genes are located on chromosome six, encoding proteins with various properties and functions [1]. Any change in either the short (p) or long (q) arm of chromosome six can result in a variety of disorders, including transient infantile diabetes mellitus, cancers, and alterations in normal development and mental function. In this regard, chromosome 6q27 encodes genes that are responsible for brain development, and the deletion of this gene leads to structural brain abnormalities with diverse phenotypic presentations [2]. Subtelomeric or terminal deletions of the chromosome 6q27 have attracted a wide range of interest due to their association with significant intellectual disabilities in children [3]. Terminal 6q deletion syndrome is a rare syndrome and only 73 cases have been reported in the literature [4]. Here, we report a pediatric case presenting with a seizure in which chromosome 6q27 terminal deletion was found following karyotyping.

## Case presentation

A two-year-old female child presented in our ED with a history of sudden onset generalized tonic-clonic seizure. The seizure lasted for approximately five mins, after which the patient responded verbally. On presentation, she was in a postictal state of drowsiness. The mother denied any history of fever, cough, runny nose, head trauma, substance ingestion, and any contact with sick individuals, or recent travel.

The patient was a full-term baby born out of nonconsanguineous marriage to a 34-year-old mother at G6P3 (three first-trimester spontaneous abortions). At birth, the facial asymmetry with right depressor angularis oris hypoplasia and a sacral dimple were noted. 

**Figure 1:**
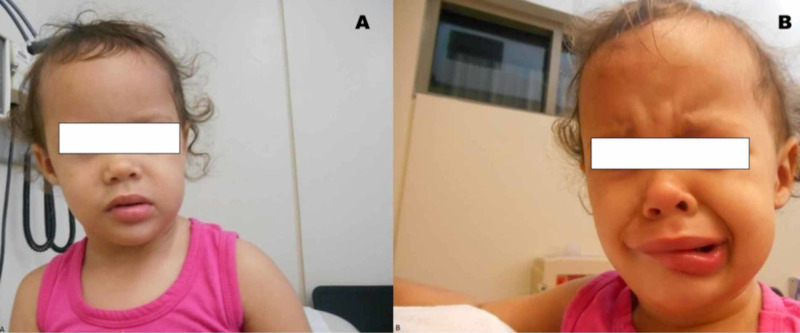
Absence of facial asymmetry at rest (A) and apparent facial asymmetry while crying (B).

On physical examination, she was well appearing and not in acute distress. There were no neurocutaneous stigmata. She had the normal ear and oral mucosa without cleft lip/palate or neck supple. Upon crying, asymmetry of the face was noted with an apparent deviation of the lower lip to the left side (Figure 1). There was a palpable thinning of the right lower lip near its right margin. Her joints were hypermobile with a decreased tone, causing inward bowing of the ankles. There was mild torticollis that resulted in a right-sided head-tilt. On neurological examination, she responded to pain and verbal commands. All the cranial nerves were intact, including the facial nerve, as determined by symmetrical frowning of the forehead, wrinkling, eye closure, nasolabial fold depth, and tearing. The general sensation and deep tendon reflexes were intact. Cardiovascular, respiratory, and abdominal examinations yielded no abnormal findings. She was able to wave goodbye or point at objects and maintained good eye contact. However, she did not feed with a spoon, imitate circular scribbles, and engage in pretend play. She knew a few words but was unable to combine them.

**Figure 2:**
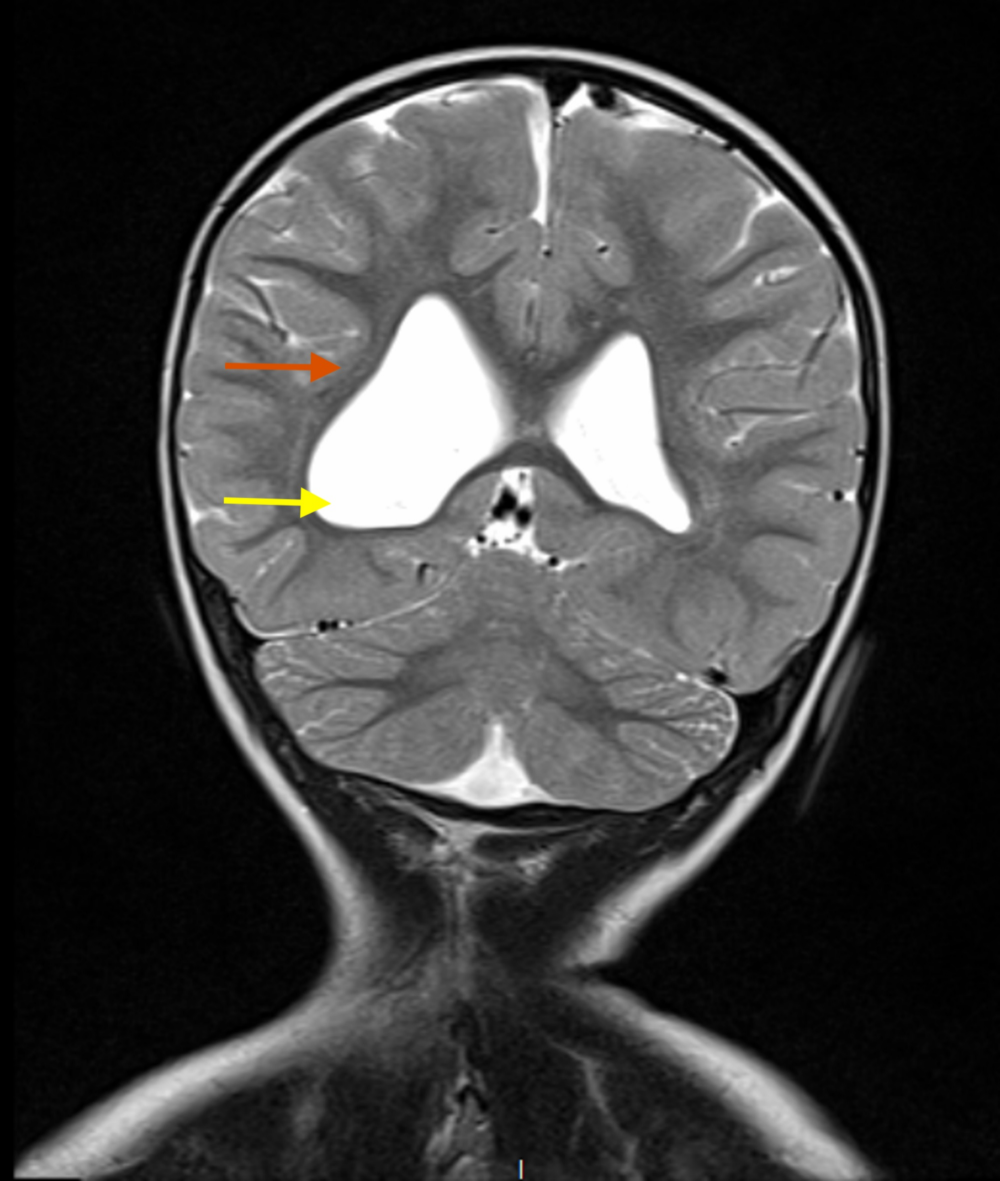
MRI of the head showing asymmetrical enlargement of the right lateral ventricle with predominant expansion of the posterior portion of the lateral ventricular body, the trigone and right occipital horn (yellow arrow), and white matter volume loss with marked thinning of white matter more pronounced in right side (red arrow).

Her complete blood count and comprehensive metabolic panel were within normal limits. The electroencephalogram demonstrated regular waves; however, MRI of the head showed asymmetrical enlargement of the right lateral ventricle with the predominant expansion of the posterior portion of the lateral ventricular body, the trigone and right occipital horn, and white matter volume loss with marked thinning of white matter more pronounced in the right side (Figure 2). These MRI findings were suggestive of long-standing chronic sequela of early hypoxic/ischemic insults with subsequent periventricular leukomalacia in the pre or perinatal period. There was no previous MRI scan for the comparison. Following karyotyping, the chromosomes exhibited normal G-band patterns within the limits of standard cytogenetic analysis and a normal female karyotype. The genetic evaluation showed a loss of approximately 783 kb of the 6Q27 terminus, and a diagnosis of 6q27 terminal deletion was made.

The patient was given levetiracetam (100 mg) intravenous. Neuroconsultation was performed, and the patient was discharged with oral levetiracetam on the third day. On the subsequent follow-up one week later, the patient was stable without any episodes of seizure. The patient has been doing well on the maintenance dosage of levetiracetam (10 mg/kg twice daily) and has remained seizure-free for six months. She is receiving early intervention services of occupational, physical, and speech therapy five times per week. 

## Discussion

Isolated terminal chromosome 6q deletion syndrome is an uncommon genetic abnormality. Patients with chromosome 6q deletion present with an array of structural and developmental anomalies, i.e., i. craniofacial dysmorphisms, like thin upper lips, downturned mouth, and abnormal ears, ii. cranial malformations, like the agenesis of the corpus callosum, periventricular nodular heterotopia, polymicrogyria, hydrocephalus, and cerebellar deformities, iii. organ dysfunctions, like muscular weakness, seizure, anosmia, cardiac abnormalities, retinal dysfunction, and vertebral defects, and iv. learning or intellectual disabilities [2, 5-11]. In our case, the patient presented with seizures, but she was found to have generalized hypotonia, developmental delay, and facial asymmetry while crying. Her facial nerve function was intact, and the facial asymmetry was attributed to the hypoplasia of the right depressor angularis oris. Similar to our case, Elia et al. have described five cases of chromosome 6q terminal deletion with seizures [9].

Terminal 6q deletion syndrome can resemble other genetic syndromes that can present with congenital seizures or facial nerve paralysis. Congenital seizure and facial paralysis can result from developmental defects or birth trauma, albeit other genetic conditions such as Möbius syndrome, Goldenhar syndrome, and Fragile X syndrome could mimic 6q27 deletion [12-14]. A congenital facial nerve paralysis is typically present in Möbius syndrome [12]. Goldenhar syndrome can have congenital facial paralysis with hemifacial microsomia and a spectrum of congenital malformations involving the structures derived from the first and second branchial arch [13]. Hypotonia, seizures, and mild to severe intellectual disabilities are possible in patients with Fragile X syndrome [14]. Fluorescent in situ hybridization (FISH) and microarrays can be employed for genetic testing and diagnostic confirmation [7, 15-16]. In our case, we performed a genetic microarray, which showed a loss of approximately 783 kb of the chromosome 6Q27 terminus. 

There is no definitive management protocol for patients with chromosome 6q27 deletion, and symptomatic and supportive management rests on the associated abnormality. Treatment and prognosis are variable, depending on the structural and functional abnormalities. In our case, we managed the patient with anti-seizure medication as she presented with a seizure. 

The primary care physicians and pediatricians should be aware of the distinctive phenotype or clinical picture to have a high degree of vigilance [17]. We recommend genetic testing of the patients and their parents in all high-index cases. Timely referral for occupational and speech therapy and special school support is required to improve functional outcomes. Early intervention improves cognitive function, slows systematic and metabolic manifestation, improves seizure control, and decreases subsequent costs and potential distress to the families [4].

## Conclusions

The terminal chromosome 6q27 deletion is a rare genomic condition that can result in intellectual disability, facial dysmorphism, and organ dysfunctions. Management of this syndrome rests on the symptomatic and supportive therapy. It is crucial to have a high clinical vigilance for early diagnosis to improve cognitive function and slow systematic and/or metabolic complications.
